# Balance between maternal antiviral response and placental transfer of protection in gestational SARS-CoV-2 infection

**DOI:** 10.1172/jci.insight.167140

**Published:** 2023-09-08

**Authors:** Juliana Gonçalves, Magda Melro, Marta Alenquer, Catarina Araújo, Júlia Castro-Neves, Daniela Amaral-Silva, Filipe Ferreira, José S. Ramalho, Nádia Charepe, Fátima Serrano, Carlos Pontinha, Maria João Amorim, Helena Soares

**Affiliations:** 1 Human Immunobiology and Pathogenesis Laboratory, iNOVA4Health, Nova Medical School, Faculty of Medical Sciences, Nova University, Lisbon, Portugal.; 2 Cell Biology of Viral Infection Lab, Gulbenkian Institute of Science, Oeiras, Portugal.; 3 Católica Biomedical Research Centre, Católica Medical School, Portuguese Catholic University, Lisbon, Portugal.; 4 Centro Hospitalar Universitário Lisboa Central, Lisbon, Portugal.; 5 iNOVA4Health, and; 6 CHRC, Nova Medical School, Faculty of Medical Sciences, Nova University, Lisbon, Portugal.

**Keywords:** COVID-19, Immunology, Immunoglobulins

## Abstract

The intricate interplay between maternal immune response to SARS-CoV-2 and the transfer of protective factors to the fetus remains unclear. By analyzing mother-neonate dyads from second and third trimester SARS-CoV-2 infections, our study shows that neutralizing antibodies (NAbs) are infrequently detected in cord blood. We uncovered that this is due to impaired IgG-NAb placental transfer in symptomatic infection and to the predominance of maternal SARS-CoV-2 NAbs of the IgA and IgM isotypes, which are prevented from crossing the placenta. Crucially, the balance between maternal antiviral response and transplacental transfer of IgG-NAbs appears to hinge on IL-6 and IL-10 produced in response to SARS-CoV-2 infection. In addition, asymptomatic maternal infection was associated with expansion of anti–SARS-CoV-2 IgM and NK cell frequency. Our findings identify a protective role for IgA/IgM-NAbs in gestational SARS-CoV-2 infection and open the possibility that the maternal immune response to SARS-CoV-2 infection might benefit the neonate in 2 ways, first by skewing maternal immune response toward immediate viral clearance, and second by endowing the neonate with protective mechanisms to curtail horizontal viral transmission in the critical postnatal period, via the priming of IgA/IgM-NAbs to be transferred by the breast milk and via NK cell expansion in the neonate.

## Graphical abstract

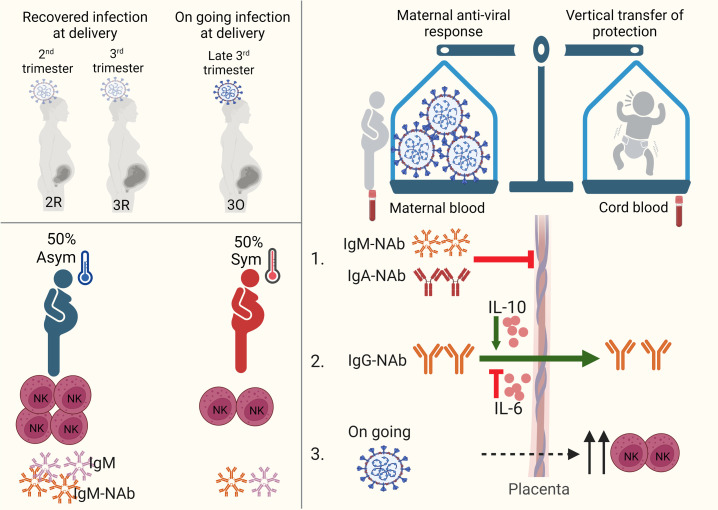

## Introduction

The maternal immune response protects the growing fetus from the vertical transmission of harmful pathogens in 2 ways: by promptly clearing the infection and via transplacental transfer of protective immune components to the fetus. This vertical transfer of protection continues after birth through secretory IgA and IgM antibodies and immune cells contained in breast milk (1–3). The outcomes of SARS-CoV-2 infection in pregnancy vary from asymptomatic or mildly symptomatic to severe disease (4–6), with increased levels of inflammatory cytokines and immune cells being detected in maternal and cord blood samples (7, 8). How cellular and humoral immune mediators balance maternal anti–SARS-CoV-2 responses with the transplacental transfer of protection to the offspring immediately via the placenta or posteriorly via breast milk remains to be addressed.

Neutralizing IgG antibodies targeting the spike protein receptor-binding domain (RBD) have been shown to play an important role in controlling SARS-CoV-2 infection (9–11). While most of the studies have focused on neutralizing antibodies (NAbs) of the IgG isotype (NAb-IgG) (9–11), recent work in COVID-19–recovered individuals have put forward that SARS-CoV-2 neutralization is associated not only with anti-RBD IgG but to a significant extent to anti-RBD antibodies of IgA and IgM isotypes (12–15). Even though IgM is commonly associated with short-lived low-affinity antibodies without neutralizing capability, potent IgM neutralizing responses have been identified to be elicited in response to viral and protozoan infections in humans (16, 17). Pregnancy is marked by changes in B cell lymphopoiesis and differentiation (18), which can potentially impact the distribution of NAb isotypes. Therefore, the individual contributions of IgM, IgA, and IgG to viral neutralization might differ in gestational infections. In this regard, a recent study found that in a cohort of Zika virus–infected (ZIKV-infected) pregnant women, IgM antibodies play a major role in viral neutralization, in an isotype-specific manner (16). Moreover, the isotypes of the NAbs condition their ability to be transported across the placenta. While IgG antibodies are transferred across the placenta beginning on gestational week 13 via the neonatal Fc receptor (FcRn) expressed on syncytiotrophoblasts (19), maternal IgM and IgA antibodies are largely prevented from being transferred to the fetus due to the lack of specific transporters in the placenta (20). Thus, the identification of the NAb isotypes in response to gestational SARS-CoV-2 infection could expand our understanding of how maternal immune responses protect the growing fetus.

In addition to antibodies, immune cells such as CD4^+^ T (21) and NK cells (22) together with the cytokines they produce also contribute to the resolution of SARS-CoV-2 infection. Namely, NK cells constitute 20% of lung lymphocytes and play an important role in eliminating SARS-CoV-2–infected cells (22). Pregnancy leads to a decrease in CD4^+^ T and NK cell levels in circulation (23). Nevertheless, increased frequency of NK cells has been detected in neonates born to pregnant women with ongoing SARS-CoV-2 infection (8). Furthermore, neonatal NK cells have been shown to display antiviral responses from birth (24). In COVID-19 patients, the profile of inflammatory cytokine responses has been associated with disease outcomes. While an interferon response is conducive to viral clearance (25), the enduring production of inflammatory cytokines IL-1β, IL-6, IL-8, IL-10, and IL-18 has been linked to disease severity (26, 27). Pregnant women have been described to mount a mild inflammatory response to SARS-CoV-2 infection that is reflected in the fetus, even in the absence of vertical viral transmission, as previously observed for gestational HIV-1 and HBV infections (7, 8, 28, 29). In view of the potential long-term harm to the developing fetus (8), it is important to determine the duration of cytokine persistence, the extent that they are transferred to the fetus, how they impinge on the transplacental transfer of protective antibodies, and to further document possible fetal immune priming.

Here, we expanded the profile of NAbs in maternal SARS-CoV-2 infection by identifying IgA and IgM NAbs and advance a possible cellular and inflammatory mechanism underpinning the balance between maternal antiviral response and vertical transfer of immune protection.

## Results

### Population.

**Figure 1:**
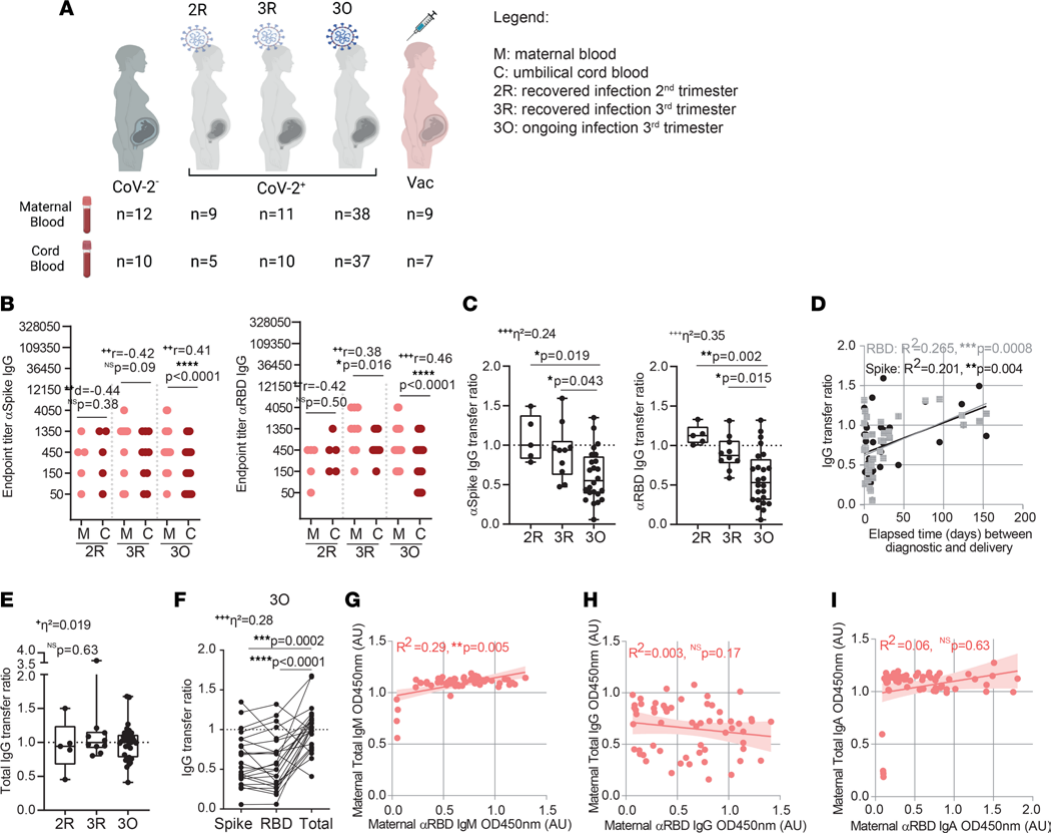
Anti–SARS-CoV-2 antibody production and transplacental transfer efficiency in gestational SARS-CoV-2 infections. (**A**) Outline of participant recruitment, divided into pregnant women negative for SARS-CoV-2 (CoV-2^–^), recovered from infection in second (2R) or third (3R) trimesters, with ongoing infection (3O), or receiving COVID-19 vaccine (Vac). (**B**) Anti-spike and anti-RBD IgG endpoint titers (*n* = 50 dyads). (**C**) Anti-spike and anti-RBD IgG transfer ratios (*n* = 39). (**D**) Correlation between anti-spike and anti-RBD IgG transfer ratio and elapsed time between diagnosis and delivery (*n* = 39). (**E**) Total IgG transfer ratios (*n* = 50). (**F**) Anti-spike, anti-RBD, and total IgG transfer ratios (*n* = 24). (**G**) Correlation between total IgM and anti-RBD IgM antibody levels (*n* = 58). (**H**) Correlation between total IgG and anti-RBD IgG antibody levels (*n* = 58). (**I**) Correlation between total IgA and anti-RBD IgA antibody levels (*n* = 58). Data represent mean ± SD for parametric tests, or median ± IQR for nonparametric tests. NS, not significant. Significance determined by parametric paired, 2-tailed *t* test (**B**), nonparametric paired Wilcoxon’s test (**B**), ordinary ANOVA with post hoc Holm-Šidák (**C** and **F**), Kruskal-Wallis with post hoc Dunn’s (**E**), Pearson’s correlation (**D**), and Spearman’s correlation (**G**–**I**). Effect sizes were determined by Cohen’s *d* (**B**), correlation coefficient *r* (**B**), and η^2^ (**C**, **E**, and **F**).

Our study consisted of 72 pregnant women recruited from May, 2020 to February, 2021, before the introduction of COVID-19 vaccination to the general population. Sixty pregnant women tested positive (CoV-2^+^; Supplemental Table 1; supplemental material available online with this article; https://doi.org/10.1172/jci.insight.167140DS1) and 12 tested negative (CoV-2^–^; Supplemental Table 2) for SARS-CoV-2 by PCR on nasopharyngeal swabs (Figure 1A). We divided the 60 CoV-2^+^ pregnant women according to gestational age at the time of infection. In the recovered second trimester (2R) group were the ones who tested positive between 85 and 154 days before delivery. The recovered third trimester (3R) group consisted of the ones who tested positive between 20 and 77 days before delivery. The ongoing (3O) group included the ones who tested positive within 11 days of delivery (Figure 1A). Pregnant women were mandatorily tested for SARS-CoV-2 by PCR in the context of monthly health provision, which allowed us to document both the time of viral infection and resolution. Fifty percent of pregnant women were symptomatic, with 4 pregnant women being admitted to the hospital due to COVID-19 symptomatology, and noninvasive oxygen support was provided to other 3. Within the CoV-2^+^ population, 50 mother–umbilical cord matched samples were collected at the time of birth, and in 82% of the deliveries a nasopharyngeal swab was performed on the newborn, with 3 babies testing positive but remaining asymptomatic and 1 baby who tested negative upon being admitted to the neonatal ICU (Supplemental Table 1). An additional 9 pregnant women were recruited between July, 2021 and February, 2022, having undergone COVID-19 mRNA BNT162b2 vaccination. Maternal and cord blood samples were collected from 6 dyads (Figure 1A and Supplemental Table 3).

### Poor neonatal NAb titers are associated with maternal production of IgM-NAbs and with impaired transplacental transfer of IgG-NAbs in symptomatic infections.

**Figure 2:**
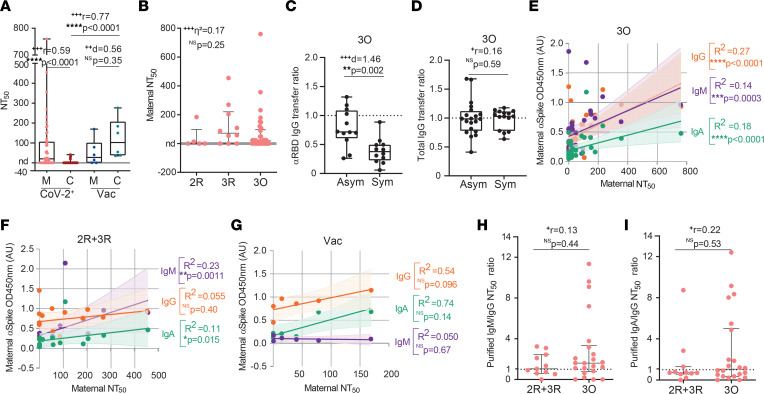
Evaluation of NAb isotypes in gestational SARS-CoV-2 infections. (**A**) NT_50_ values for maternal and cord blood paired samples from SARS-CoV-2–infected (CoV-2^+^; *n* = 50 dyads) and vaccinated mothers (Vac, *n* = 6 dyads). (**B**) NT_50_ for CoV-2^+^ maternal samples segregated by gestational age of infection (*n* = 50). (**C**) Anti-RBD IgG transfer ratio in the presence (Sym) or absence (Asym) of symptoms in ongoing maternal infections (3O, *n* = 24). (**D**) Total IgG transfer ratio in the presence (Sym) or absence (Asym) of symptoms in ongoing maternal infections (3O, *n* = 35). (**E**) Correlation between maternal anti-spike IgA, IgG, and IgM and NT_50_ in ongoing infections (3O, *n* = 35). (**F**) Correlation between maternal anti-spike IgA, IgG, and IgM and NT_50_ in recovered infections (2R + 3R, *n* = 15). (**G**) Correlation between maternal anti-spike IgA, IgG, and IgM and NT_50_ in vaccinated participants (Vac, *n* = 6). (**H**) Ratios of NT_50_ values obtained from purified IgM and IgG fractions in maternal infections (*n* = 33). (**I**) Ratios of NT_50_ values obtained from purified IgA and IgG fractions in maternal infections (*n* = 33). Data represent mean ± SD for parametric tests, or median ± IQR for nonparametric tests. nd, not detectable. NS, not significant. Significance determined by parametric paired, 2-tailed *t* test (**A**), unpaired, 2-tailed *t* test (**C**), nonparametric paired Wilcoxon’s test (**A**), Mann-Whitney test (**A**, **D**, **H**, and **I**), Kruskal-Wallis with post hoc Dunn’s (**B**), Spearman’s correlation (**E**–**G**), and Pearson’s correlation (**F** and **G**). Effect sizes were determined by Cohen’s *d* (**A** and **C**), correlation coefficient *r* (**A**, **D**, **H**, and **I**), and η^2^ (**B**).

**Figure 3:**
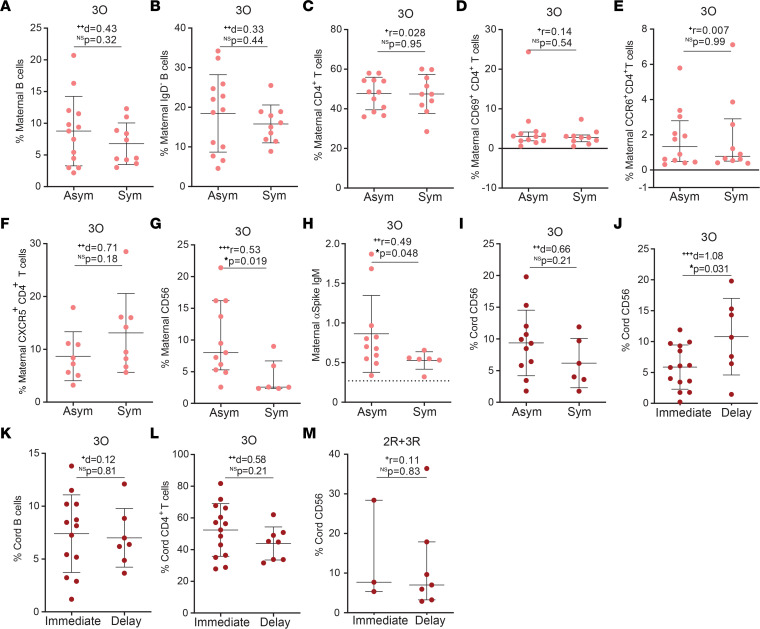
Cellular and humoral immune responses in symptomatic and asymptomatic gestational SARS-CoV-2 infection. (**A**) Maternal B cell frequency in ongoing SARS-CoV-2 (3O) infection in function of the presence (Sym) or absence (Asym) of symptoms (*n* = 23). (**B**) Frequency of IgD^–^ B cells as in **A** (*n* = 23). (**C**) Frequency of CD4^+^ T cells as in **A** (*n* = 22). (**D**) Frequency of CD69^+^CD4^+^ T cells as in **A** (*n* = 22). (**E**) Frequency of CCR6^+^CD4^+^ T cell as in **A** (*n* = 22). (**F**) Frequency of CXCR5^+^CD4^+^ T cell as in **A** (*n* = 16). (**G**) Maternal NK cell frequency in participants diagnosed for SARS-CoV-2 within 7 days of delivery in asymptomatic (Asym) and symptomatic (Sym) individuals (*n* = 17). (**H**) Maternal anti-spike IgM levels as in **G**. (**I**) Cord blood NK cell frequency from participants diagnosed for SARS-CoV-2 within 7 days of delivery, segregated by the presence (Sym) or absence (Asym) of maternal symptoms (*n* = 17). (**J**) Cord blood NK cell frequency upon either immediate or delayed umbilical cord clamping, in ongoing maternal infections (3O, *n* = 21). (**K**) Cord blood B cell frequency as in **J** (*n* = 20). (**L**) Cord blood CD4^+^ T cell frequency as in **J** (*n* = 22). (**M**) Cord blood NK cell frequency upon either immediate or delayed umbilical cord clamping, in second and third trimester recovered (2R + 3R) infection (*n* = 10). Data represent mean ± SD for parametric tests, or median ± IQR for nonparametric tests. NS, not significant. Significance determined by unpaired, 2-tailed *t* test (**A**–**C**, **F**, and **I**–**L**) and Mann-Whitney test (**D**, **E**, **G**, **H**, and **M**). Effect sizes were determined by Cohen’s *d* (**A**–**C**, **F**, and **I**–**L**) and correlation coefficient *r* (**D**, **E**, **G**, **H**, and **M**).

The transfer of antibodies from maternal to fetal circulation relies on the FcRn expressed on the placenta. Of the antibody isotypes produced in response to viral infection, only IgG, but not IgA nor IgM, are transported across the placenta via the FcRn (19). Hence, detection of IgA and IgM antibodies in cord blood is used as diagnostic for fetal infection (30). Out of 50 dyads, we detected only one case where both anti-spike IgA and IgM antibodies were present in cord blood (Supplemental Figure 1, A and B). Anti-spike IgG was found in all mothers and neonates born to CoV-2^+^ mothers in the 2R and 3R groups and in 68.4% of mothers and 56.8% of neonates born to CoV-2^+^ mothers in the 3O group (Figure 1B). In recovered SARS-CoV-2 maternal infection, anti-spike and anti-RBD titers (Figure 1B; 2R and 3R) remained overall comparable within mother-neonate dyads. In contrast, there was a significant decrease in anti-spike/RBD IgG titers in neonates born to mothers with ongoing SARS-CoV-2 infection (Figure 1B; 3O). In fact, the transfer ratios of anti-spike and anti-RBD IgG antibodies were approximately 1 in 2R and 3R groups and approximately 0.5 in the 3O group (Figure 1C), with the efficiency of antibody transfer increasing with time elapsed between SARS-CoV-2 infection and delivery (Figure 1D). To evaluate whether the decrease in anti-spike/RBD antibody transfer in ongoing infection was due to a saturation of the FcRn (31–34), we assessed the transfer ratio of total IgG, which we found to be approximately 1 in all analyzed groups, including 3O (Figure 1E). Moreover, the total IgG transfer ratio was also significantly higher than the ratio for anti-spike/RBD for all dyads analyzed except for 3 dyads, which were associated with premature deliveries (Figure 1F). As expected, total IgA, IgG, and IgM remained constant, regardless of gestational age of infection (Supplemental Figure 1C) and we found that only the production of anti-RBD IgM antibodies was positively associated with total IgM levels (Figure 1, G–I), albeit with a modest *R*^2^ value.

We then addressed whether the sex of the fetus had an impact on the immune response to infection. We did not observe changes in maternal production of anti-spike IgA, IgM, or IgG antibodies in function of the sex of the fetus (Supplemental Figure 1D). However, in dyads with ongoing maternal SARS-CoV-2 infection, we observed a significant decrease in anti-spike IgG levels in male neonates, which was not observed in female neonates (Supplemental Figure 1, E and F).

Next, we compared the capacity of maternal and neonatal anti-spike antibodies to neutralize viral entry. Following SARS-CoV-2 infection, only 58.6% of mothers and 10% of neonates possessed NAbs (Figure 2A and Supplemental Figure 2, A and B; CoV-2^+^), with the frequency of detection of maternal NAbs (Figure 2B) decreasing with time elapsed since infection from approximately 60% in the 3R and 3O groups to 44.4% in the 2R group, suggesting a possible waning of NAbs in infections occurring in the second trimester. Of the 50 dyads tested, we could only detect NAbs in 5 neonates (IQR: 23–40; Figure 2A and Supplemental Figure 2B; CoV-2^+^). In contrast, in vaccinated dyads, NAbs were present in all neonatal blood samples and their titers were similar to their maternal counterparts for each dyad (Figure 2A and Supplemental Figure 2, A and B; Vac). Infected and vaccinated mothers had equivalent neutralization titers (Figure 2A), making it unlikely that the near absence of NAbs in neonates was primarily due to failure of the infection to induce NAbs. In contrast to what we had found for anti-spike IgG transplacental transfer (Figure 1D), the transfer ratio of NAbs did not increase with time elapsed since infection (Supplemental Figure 2C). As one of the factors known to hinder IgG transplacental transfer is inflammation (9), we next explored how the presence/absence of symptoms affected NAb transplacental transfer. As the RBD is particularly enriched in neutralizing epitopes (9–11), we used anti-RBD antibodies as an approximation to gain insight into how the presence/absence of symptoms would affect NAb transplacental transfer. The presence of symptoms did not affect the maternal levels of anti-RBD, nor of NAbs, including in study participants with ongoing infection (Supplemental Figure 2, D–G). Crucially, the transfer of anti-RBD (Figure 2C), but not of total IgG (Figure 2D), plummeted by approximately 40% in symptomatic ongoing maternal infection.

Anti-spike IgM and IgA have been associated with SARS-CoV-2 neutralization in both COVID-19 disease and vaccination (12–14). This raises the possibility that poor neutralizing response in neonates might result from maternal neutralizing activity being, at least partly, mediated by IgM/IgA and thus not transferrable to the neonate. To address this hypothesis, we correlated the levels of maternal anti-spike IgA, IgG, and IgM antibody with neutralization titers in ongoing infection and recovered groups. In ongoing infection, neutralization titers correlated with all 3 anti-spike antibody isotypes (Figure 2E), at moderate *R*^2^ values. Furthermore, neutralization was stronger in mothers who displayed both IgG and IgM anti-spike antibodies, compared with cases that only presented anti-spike IgG or anti-spike IgM (Supplemental Figure 2, H and I). Intriguingly, in recovered maternal infection, NAb titers correlated at modest *R*^2^ values with anti-spike IgA and IgM, but not with anti-spike IgG (Figure 2F; 2R + 3R). Moreover, the presence of anti-spike IgG antibodies alone was insufficient to enact neutralization (Supplemental Figure 2, H and I). In contrast, in COVID-19–vaccinated women, neutralization appears to be associated with anti-spike IgG, but not with anti-spike IgM or IgA antibodies (Figure 2G), which is in agreement with the effective transfer to the neonates of NAbs that we (Figure 2A; Vac) and others (35–37) have observed. To investigate whether maternal SARS-CoV-2 infection elicited NAbs of the IgA or IgM isotypes, we purified IgG, IgA, and IgM fractions (Supplemental Figure 2J) and repeated the neutralization assays. All antibody fractions possessed neutralizing activity (Figure 2, H and I). We found that in 16 out of 22 participants with ongoing infection, IgM antibodies exhibited up to 12-fold higher neutralization than IgG (Figure 2H). Moreover, in ongoing infections, half of the IgA fractions were more neutralizing than the IgG ones (Figure 2I). In recovered infections, all antibody isotypes, IgG, IgM, and IgA, displayed similar neutralizing capacities (Figure 2, H and I).

These results indicate that sparse detection of NAbs in neonates is due to combined action of symptomatic infection particularly hindering the placental transfer of anti-RBD IgG and of a skewing of maternal NAb production toward IgA and IgM isotypes, which do not cross the placenta, in response to SARS-CoV-2 infection.

### Asymptomatic maternal infection is characterized by enhanced early immune responses.

To identify putative immune markers of infection outcomes, we analyzed maternal and neonatal cellular responses in asymptomatic versus symptomatic ongoing maternal infections. As expected from the short interval spanning between infection and sample collection, the maternal frequency of B cells, class-switched IgD^–^ B cells, CD4^+^ T cells, activated CD4^+^ T cells, CCR6^+^ T cells, and CXCR5^+^ T cells remained equivalent in the presence or absence of symptoms (Figure 3, A–F, and Supplemental Figure 3, A and B). Interestingly, asymptomatic ongoing infection gave rise to higher frequency of NK cells in maternal circulation (Figure 3G and Supplemental Figure 3C). The implication that early immune responses might be important to curtail infection prompted us to look at IgM antibodies, and we found that anti-spike IgM levels were higher in asymptomatic ongoing infections (Figure 3H). Although the presence of maternal symptoms did not significantly affect NK cell frequency in the cord blood (Figure 3I), we observed that delayed umbilical cord clamping led to higher frequency of NK cells in neonates born to mothers with ongoing infection (Figure 3J). This enrichment in NK cell frequency in the cord blood appears to be specific and not a simple result of increased blood influx, as delayed clamping did not alter the neonatal frequency of B cells (Figure 3K) or CD4^+^ T cells (Figure 3L). Similarly, delayed clamping had no effect on neonatal NK cell frequency in resolved SARS-CoV-2 infections (Figure 3M).

Altogether, our results indicate that an early immune response conveyed by NK cells, and to a lesser extent by anti-spike IgM, is associated with asymptomatic maternal infection.

### Cellular and cytokine immune responses differ in gestational age–matched infected or vaccinated mothers.

**Figure 4:**
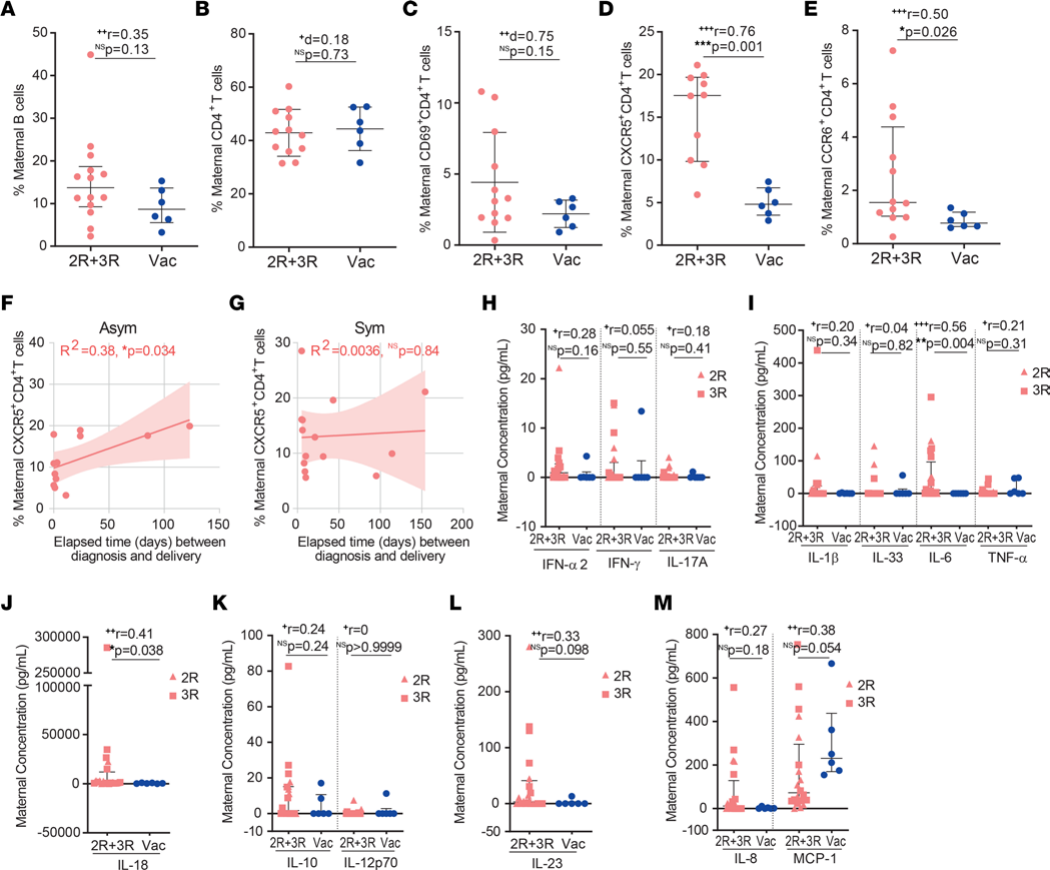
Cellular and cytokine responses in gestational age–matched SARS-CoV-2 infection and COVID-19 vaccination. (**A**) Frequency of circulating B cells in SARS-CoV-2 recovered (2R + 3R, *n* = 14) and in vaccinated participants (Vac, *n* = 6). (**B**) Frequency of CD4^+^ T cells as in **A** (2R + 3R, *n* = 12; Vac, *n* = 6). (**C**) Frequency of CD69^+^CD4^+^ T cells as in **A** (2R + 3R, *n* = 12; Vac, *n* = 6). (**D**) Frequency of CXCR5^+^CD4^+^ T cells as in **A** (2R + 3R, *n* = 10; Vac, *n* = 6). (**E**) Frequency of CCR6^+^CD4^+^ T cells as in **A** (2R + 3R, *n* = 12; Vac, *n* = 6). (**F** and **G**) Correlation between the frequency of maternal CXCR5^+^CD4^+^ T cells and the elapsed time between diagnosis and delivery in asymptomatic (**F**, Asym; *n* = 12) and symptomatic (**G**, Sym; *n* = 14) individuals. (**H**–**M**) Maternal plasma concentration (pg/mL) of (**H**) IFN-α2, IFN-γ, IL-17A; (**I**) IL-1β, IL-33, IL-6, TNF-α; (**J**) IL-18; (**K**) IL-10, IL-12p70; (**L**) IL-23; and (**M**) IL-8 and MCP-1 in recovered gestational SARS-CoV-2 infection (2R + 3R, *n* = 20) and vaccinated group (Vac, *n* = 6). Data represent mean ± SD for parametric tests, or median ± IQR for nonparametric tests. NS, not significant. Significance determined by Mann-Whitney test (**A**, **D**, **E**, and **H**–**M**), unpaired, 2-tailed *t* test (**B** and **C**), and Pearson’s correlation (**F** and **G**). Effect sizes were determined by correlation coefficient *r* (**A**, **D**, **E**, and **H**–**M**) and Cohen’s *d* (**B** and **C**).

To pinpoint long-lasting cellular immune changes brought by SARS-CoV-2 infection, we compared the B cell and CD4^+^ T cell profiles in pregnant women who had been either infected or vaccinated at similar gestational ages. Delivery occurred at an average of 66.8 and of 73.9 days after diagnosis or after inoculation of the second vaccine dose, respectively. The frequency of B and CD4^+^ T cells was equivalent between the SARS-CoV-2–infected (2R and 3R) and COVID-19–vaccinated groups (Figure 4, A and B). When we looked in more detail at CD4^+^ T cell activation and migratory status, we observed that SARS-CoV-2 infection did not alter the CD4^+^ T cell activation state (Figure 4C) but led to an increase in the frequency of CXCR5^+^ T cells, and to a lesser extent of CCR6^+^ T cells (Figure 4, D and E). Nonetheless, this increase in the frequency of maternal CCR6^+^ and CXCR5^+^ T cells was not reflected in the fetus (Supplemental Figure 4, A and B). CXCR5^+^ T cell expansion was proportional to the time elapsed between diagnosis and delivery, in asymptomatic, but not in symptomatic, infections (Figure 4, F and G). Despite the role of CXCR5^+^ T cells in directing antibody production (38), their expansion was not associated with higher anti-spike IgG, IgA, or IgM levels (Supplemental Figure 4, C–E). Then, we compared the inflammatory profiles of participants who had been either infected or vaccinated at a similar gestational age (Figure 4, H–M). SARS-CoV-2 infection led to higher concentration of maternal IL-6 and IL-18, two cytokines that have been implicated in pregnancy complications and preterm delivery (39), when compared with vaccination (Figure 4, I and J).

SARS-CoV-2 maternal infections display higher frequencies of CXCR5^+^CD4^+^ and CCR6^+^CD4^+^ T cells and higher plasma concentrations of IL-6 and IL-18, when compared with gestational age–matched COVID-19–vaccinated counterparts.

### Maternal inflammatory response balances antibody placental transfer with NK cell expansion.

**Figure 5:**
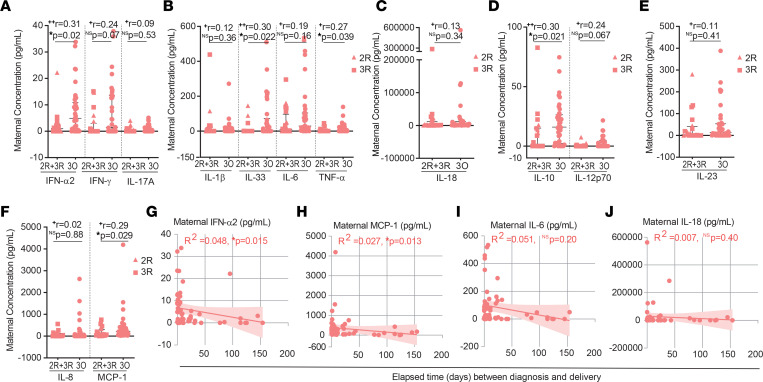
Comparison of cytokine production between recovered and ongoing SARS-CoV-2 gestational infection. (**A**–**F**) Maternal plasma concentration (pg/mL) of (**A**) IFN-α2, IFN-γ, IL-17A; (**B**) IL-1β, IL-33, IL-6, TNF-α; (**C**) IL-18; (**D**) IL-10, IL-12p70; (**E**) IL-23; and (**F**) IL-8 and MCP-1 in recovered (2R + 3R, *n* = 20) and ongoing (3O, *n* = 38) gestational SARS-CoV-2 infection. (**G**–**J**) Correlation between maternal concentration of (**G**) IFN-α2; (**H**) MCP-1; (**I**) IL-6; and (**J**) IL-18 and elapsed time between diagnosis and delivery (*n* = 58). Data represent median ± IQR for nonparametric tests. NS, not significant. Significance determined by Mann-Whitney test (**A**–**F**) and Spearman’s correlation (**G**–**J**). Effect sizes were determined by correlation coefficient *r* (**A**–**F**).

**Figure 6:**
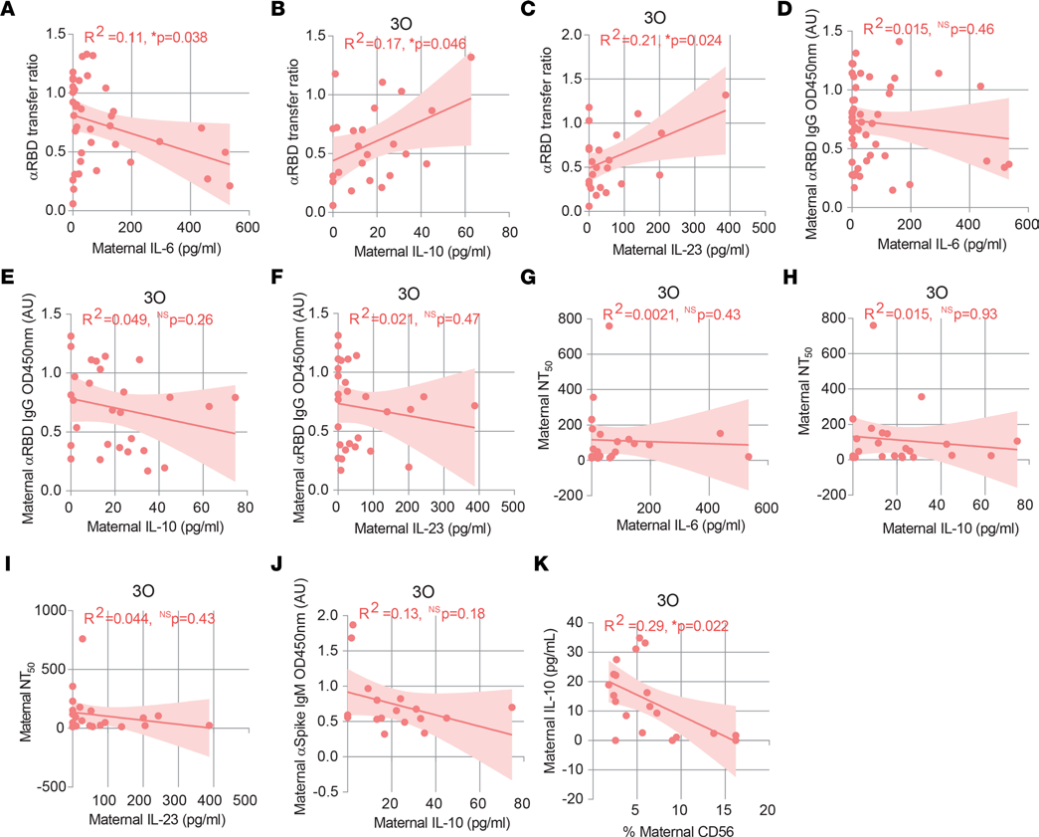
Association between cytokine production and cellular and humoral immune responses in gestational SARS-CoV-2 infection. (**A**) Correlation between anti-RBD IgG transfer ratio and IL-6 concentration in CoV-2^+^ maternal plasma (*n* = 39). (**B**) Correlation between anti-RBD IgG transfer ratio and IL-10 concentration in maternal plasma in ongoing (3O) infection (*n* = 24). (**C**) As in **B** for IL-23. (**D**) Correlation between anti-RBD IgG levels and IL-6 concentration in CoV-2^+^ maternal plasma (*n* = 48). (**E**) Correlation between anti-RBD IgG levels and IL-10 concentration in maternal plasma in ongoing (3O) infection (*n* = 28). (**F**) As in **E** for IL-23. (**G**) Correlation between NT_50_ and IL-6 concentration in maternal plasma in ongoing (3O) infection (*n* = 23). (**H**) As in **G** for IL-10. (**I**) As in **G** for IL-23. (**J**) Correlation between anti-spike IgM levels and IL-10 concentration in CoV-2^+^ maternal plasma within 7 days between diagnosis and delivery (*n* = 17). (**K**) Correlation between IL-10 concentration and NK cell frequency in ongoing (3O) infection (*n* = 20). NS, not significant. Significance determined by Pearson’s correlation (**A**–**C**, **E**, and **F**) and Spearman’s correlation (**D** and **G**–**K**).

Cytokines are key players in coordinating a healthy pregnancy (39). Nonetheless, inflammatory responses during pregnancy have been implicated in pregnancy complications (39), decreased rate of IgG transplacental transfer (19), and neurodevelopmental deficits (39). We performed a 13-cytokine multiplex assay on plasma isolated from maternal and cord blood samples. To sort out long-term from short-term cytokine changes, we started by comparing the inflammatory profile of mothers with recovered (2R + 3R) or ongoing (3O) infection. Mothers with ongoing infection exhibited higher levels of the antiviral mediator IFN-α2, the inflammatory cytokines IL-33 and TNF-α, the antiinflammatory cytokine IL-10, and the chemokine MCP-1 (Figure 5, A–F). Maternal plasma concentration of IFN-α2 and MCP-1 inversely correlated with time elapsed between infection and delivery (Figure 5, G and H), reinforcing their role in acute infection. In contrast, elevation of inflammatory IL-6 and IL-18 was independent of time of infection (Figure 5, I and J). Next, we sought to probe how the inflammatory response affected anti-RBD IgG transplacental transfer in ongoing infections. We found that anti-RBD IgG transplacental transfer inversely correlated with IL-6 (Figure 6A), but positively correlated with IL-10 and with IL-23 (Figure 6, B and C). No association was detected between IL-6, IL-10, and IL-23 concentration and anti-RBD IgG or half-maximal neutralization titer (NT_50_) levels in maternal plasma (Figure 6, D–I).

Lastly, we sought to scrutinize how the maternal inflammatory response would affect the early NK cell and IgM immune responses in asymptomatic infection (Figure 3, G–I). IL-10 inversely associated with NK cell frequency, and no association was found between IL-10 and anti-spike IgM secretion (Figure 6, J and K).

Altogether, these data suggest that SARS-CoV-2 infection can lead to durable inflammatory responses long after the infection has been cleared. Moreover, they indicate that IL-10 and IL-6 inversely associate with NK cell frequency and with the transfer of protective anti-RBD IgG to the neonate, respectively.

### Higher concentration of at least one inflammatory cytokine in the cord blood in half of mother-neonate dyads.

**Figure 7:**
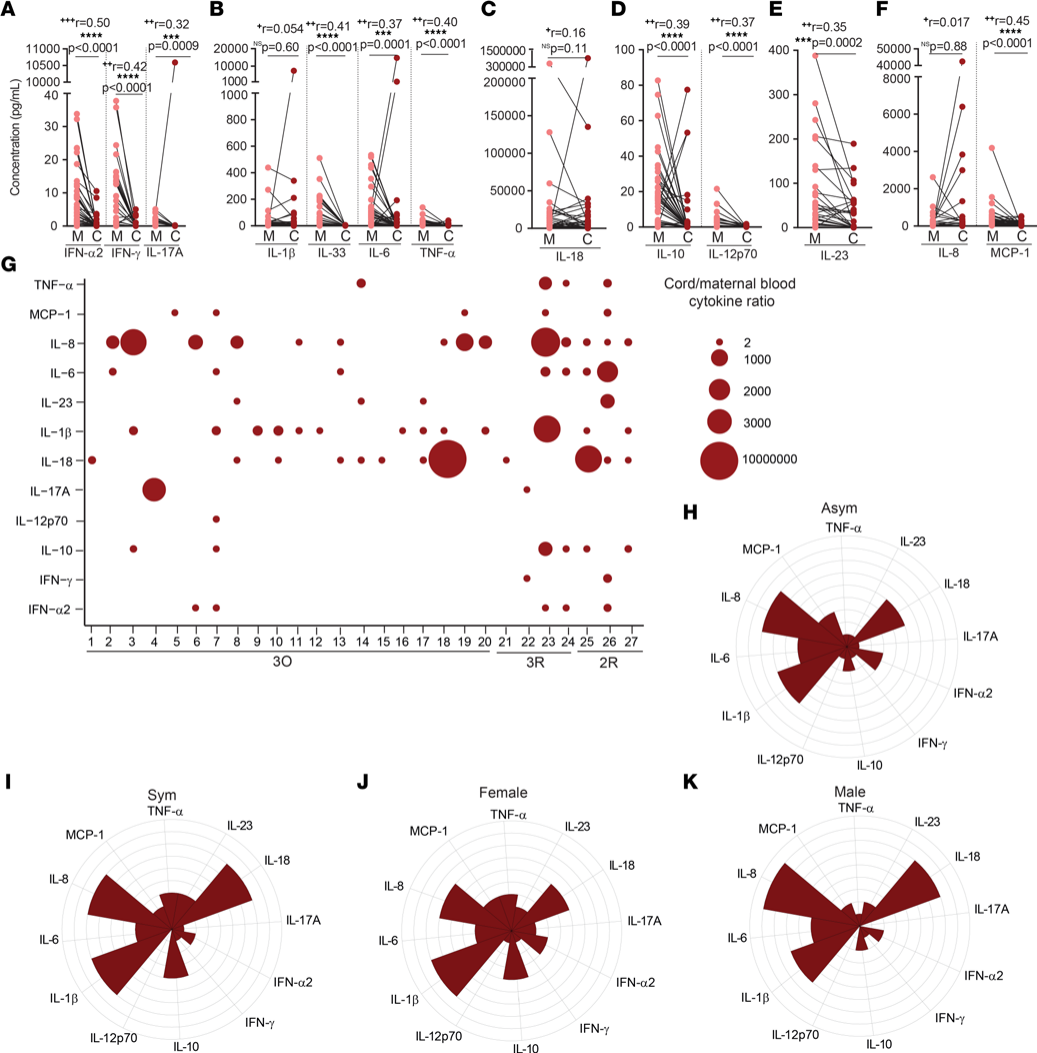
Inflammatory profile in mother-neonate dyads, upon maternal SARS-CoV-2 infection. (**A**–**F**) Plasma concentration (pg/mL) of (**A**) IFN-α2, IFN-γ, IL-17A; (**B**) IL-1β, IL-33, IL-6, TNF-α; (**C**) IL-18; (**D**) IL-10, IL-12p70; (**E**) IL-23; and (**F**) IL-8 and MCP-1 in CoV-2^+^ paired mother-neonate dyads (*n* = 49). (**G**) Ratio of cytokines in cord and maternal blood (*n* = 27). (**H** and **I**) Polar plots of cytokine profile in neonates born to (**H**) asymptomatic (Asym) and (**I**) symptomatic (Sym) mothers, in dyads with cord/maternal ratio >1.5 (*n* = 27). (**J** and **K**) Polar plots of cytokine profile of (**J**) female and (**K**) male neonates, in dyads with cord/maternal ratio >1.5 (*n* = 27). NS, not significant. Significance determined by nonparametric paired Wilcoxon’s test (**A**–**F**) and unpaired, 2-tailed *t* test (**H**–**K**). Effect sizes were determined by correlation coefficient *r* (**A**–**F**).

Even mild maternal immune responses to viral infections have resulted in adverse long-term outcomes associated with fetal inflammatory cytokine exposure (39). Of our 50 mother-neonate dyads, 27 neonates displayed higher concentration of at least one (IFN-α2, IFN-γ, IL-17, IL-1β, IL-6, TNF-α, IL-18, IL-10, IL-12, IL-23, IL-8, and/or MCP-1) inflammatory cytokine. Only IL-33 was not elevated in cord blood samples (Figure 7, A–F). In neonates born to SARS-CoV-2–infected mothers, the increase in cytokine concentration in the cord blood ranged from 2-fold to 1 × 10^7^-fold (Figure 7G). In contrast, neonates born to control noninfected and nonvaccinated mothers displayed a more contained cytokine upregulation both in terms of magnitude, ranging from 2- to approximately 7500-fold, and in terms of the cytokines upregulated, which were restricted to MCP-1, IL-8, IL-1β, and IL-10 (Supplemental Figure 5). Next, we illustrated through polar plots the possible contribution of maternal symptomatology and neonatal sex to the inflammatory profile of cord blood. In dyads with a cord/maternal ratio higher than 1.5 for at least one cytokine, there was a trend, albeit not statistically significant, for increased IFN-α2, IL-10, IL-23, and TNF-α in neonates born to symptomatic mothers (Figure 7, H and I). When segregated by neonatal sex, IFN-α2, IL-10, and TNF-α appear to be enriched in female neonates, while IL-8 and IL-18 appear to be increased in male neonates (Figure 7, J and K), although it did not reach statistical significance.

All in all, these data underscore that even asymptomatic maternal infections elicit fetal exposure to at least one inflammatory cytokine, in at least 50% of the cases.

### SARS-CoV-2 transplacental transmission.

**Figure 8:**
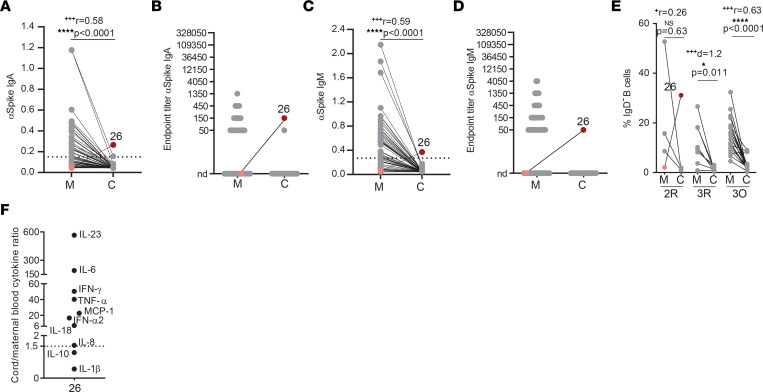
Vertical SARS-CoV-2 transmission. (**A** and **B**) Paired mother (M)–cord blood (C) dyad analysis of anti-spike IgA levels (**A**) and endpoint titers (**B**), with dyad 26 highlighted in pink and maroon (*n* = 50). (**C** and **D**) As in **A** and **B** but for IgM. (**E**) Frequency of IgD^–^ B cells in paired mother–cord blood dyads in recovered second (2R) and third (3R) trimester, or in ongoing (3O) infection, with dyad 26 highlighted in pink and in maroon (*n* = 31). (**F**) Cytokine ratio between cord and maternal blood for dyad 26. Dashed line indicates assay cutoff; nd, not detectable. NS, not significant. Significance determined by nonparametric paired Wilcoxon’s test (**A**, **C**, and **E**) and parametric paired, 2-tailed *t* test (**E**). Effect sizes were determined by correlation coefficient *r* (**A**, **C**, and **E**) and Cohen’s *d* (**E**).

SARS-CoV-2 vertical transmission is a rare event occurring in 0%–7.7% of pregnancies (7, 40, 41). Detection of IgM, and to a lesser extent of IgA, in the cord blood represents an immune fetal response to viral infection and thus is used to document vertical transmission events (19, 30). We detected anti-spike IgA and IgM in 1 (out of 50) mother-neonate dyad (Figure 8, A–D) in which maternal infection had occurred during the second trimester of gestation, a gestational period with detectable ACE2 expression (42). Maternal blood was negative for anti-spike IgA and IgM antibodies, which ruled out cross-contamination (Figure 8, A–D). Although we cannot ascertain their antigenic specificity, IgD^–^ B cells were expanded in this cord blood (Figure 8E), further supporting fetal infection. Moreover, in this dyad all but 2 cytokines were present at a higher concentration in the cord blood than in the mother (Figure 8F).

## Discussion

In this study, we investigated the cellular and molecular underpinnings regulating the balance between maternal SARS-CoV-2 clearance and transplacental transfer of protection over the course of pregnancy. We show that while antiviral cytokine production is short-term, SARS-CoV-2 gestational infection is associated with high concentrations of inflammatory cytokines, which could be detected months after the infection. Finally, we describe that even asymptomatic maternal SARS-CoV-2 infection elicits fetal exposure to several inflammatory cytokines, in a significant proportion of neonates.

In our cohort, 3 neonates tested positive for SARS-CoV-2 by PCR. However, we could not exclude that these infections occurred postnataly, rather than in utero. Indeed, umbilical cord testing for anti-spike IgM, which more accurately identifies in utero infections (30, 43), was negative in all 3 cases. Distinct from prior reports that focused on infections at the time of delivery (7, 8, 42, 44), our study encompassed SARS-CoV-2 infections spanning the second and third trimesters of gestation. This allowed us to compare in a gestational age–matched manner humoral, cellular, and inflammatory immune responses following SARS-CoV-2 infection and COVID-19 mRNA vaccination.

Vertical transfer of SARS-CoV-2 antibodies, either in utero or via breast milk, is purported to provide protection to the neonate over the first few months of life (3, 7, 8, 45–49). We observed selective impaired transplacental transfer of anti-spike IgG only in ongoing SARS-CoV-2 maternal infection, which is in line with previous findings proposing that altered anti-spike IgG glycosylation patterns are at the root of their reduced transplacental transfer (50). In contrast, second and earlier third trimester infections displayed anti-spike IgG transfer ratios of approximately 1, in the range of what has been observed for other acute viral infections (51–53). Whether this regularization of antibody transfer with time elapsed since infection is due to a reversion of anti-spike IgG glycosylation patterns (50) once inflammation is subdued, or a result of an extended window for antibody transfer to occur, or a combination of the two still needs to be addressed.

Consistent with previous studies (35–37), we observed that maternal SARS-CoV-2 infection elicited similar NAb titers to COVID-19 mRNA vaccination. Nonetheless, we noticed a decrease in the frequency of NAb detection in second trimester infections. This might be due to the NAbs waning, to a decrease in NAb production in the second, and more tolerogenic (39), trimester of pregnancy, or to a combination of the two. Distinctively from vaccination, SARS-CoV-2 maternal infection led to a near absence of NAbs in cord blood, independently of gestational age at time of infection. This was unlikely due to mismatched antibody-spike protein sequences in our neutralization assays. As at the time of recruitment (May, 2020 to February, 2021), the spike protein sequence of the SARS-CoV-2 variant prevalent in Portugal (54) coincided with the spike sequence used both in mRNA COVID-19 vaccines and in our in vitro neutralization assay. Reduced transplacental transfer NAbs upon maternal SARS-CoV-2 infection had been previously noted (55–57) but the factors that govern it were yet to be identified. We advanced on this by identifying that both maternal inflammation and the nature of the NAb response are associated with impaired NAb placental transfer.

To address the role of maternal inflammation in NAb placental transfer, we circumvented the limitation of having a very low number of cord blood samples with NAbs (10%) by using anti-RBD IgG as an approximate measure for neutralizing IgG antibodies (9–11). Our results show that symptomatic infection is associated with reduced transplacental transfer of anti-RBD IgG. Moreover, we found that elevated IL-6 was associated with lower anti-RBD IgG transfer, while elevated IL-10 and IL-23 correlated with increased anti-RBD IgG transplacental transfer. It is possible that IL-6, IL-10, and IL-23 could affect NAb transfer efficiency by altering NAb glycosylation and or FcRn-NAb binding avidity (19, 58). Next, we identified how the nature of the NAb response would impinge on NAb placental transfer, as IgA and IgM are not amenable to FcRn-mediated transplacental transfer (20). We found that viral neutralization was not restricted to IgG isotype. In fact, purified IgA and IgM were approximately 3-fold more efficient at neutralizing a SARS-CoV-2 pseudovirus than purified IgG, in ongoing infections. It is possible that in vivo IgM neutralizing activity might even be higher, as IgM has been estimated to trigger a complement response that is many-fold more effective than IgG (59). Neutralizing IgM responses have also been found for HIV (60), VSV (61), rabies virus (62), and influenza virus (63–65). In COVID-19 recovered individuals, neutralizing activity has been shown to correlate better with anti-spike IgM than with anti-spike IgG (12–14). Furthermore, depleting COVID-19 convalescent plasma from IgM or IgG resulted in a 5.5- and 4.5-fold decrease in NT_50_ (15) and purified IgM and IgG fractions displayed similar neutralizing activities in an in vitro neutralization assay (14). It is possible that changes in B cell populations brought upon by pregnancy (18) might further skew the NAb response toward IgM and IgA isotypes, magnifying the physiological relevance of IgM NAbs. Physiologically, a possible skewing toward IgM and IgA during pregnancy might serve to mitigate the risk of IgG-assisted viral transcytosis across the placenta and subsequent fetal infection (66, 67). Although the role of IgM in maternal infections has remained largely undefined, a recent study in gestational ZIKV has provided insight into its crucial protective role (16). In a cohort of ZIKV-infected pregnant women, IgM was shown to contribute to early viral neutralization. Moreover, an anti-ZIKV IgM with ultrapotent neutralization capability was isolated and its neutralizing capability was demonstrated to be isotype specific (16). Anti-spike IgM and IgG might play important and complementary roles in clearing SARS-CoV-2 infection. While IgM’s high antigen avidity may allow higher tolerance to evolving SARS-CoV-2 variants, IgGs can leverage an important protective role through multiple FcγR-mediated functional activities, beyond neutralization. Altogether, the decrease that we report here in the transplacental transfer of NAbs is likely to be multifaceted and dependent on the cohort composition. As the transfer efficiency of anti-RBD IgG decreased in symptomatic infections, it is possible that our cohort, which consisted of approximately 50% symptomatic infections, was better poised to detect possible constraints to the transplacental transfer of NAbs than previous works composed mainly of asymptomatic participants (35–37). Recruiting more diverse cohorts in terms of gestational age of infection and symptomatology is likely to afford a more complete picture of the ramifications of gestational SARS-CoV-2 infections.

It is not clear why IgG-NAbs produced in response to maternal SARS-CoV-2 infection displayed lower transfer ratios than those produced upon COVID-19 vaccination. It is possible that infection and vaccination lead to the production of distinct IgG subclass profiles with different binding affinities for FcRn (68, 69) and/or to other FcRs expressed in the placenta (31). Altered cytokine profiles in symptomatic infections might decrease anti-RBD IgG transplacental transfer by altering its glycosylation profiles, as previously reported (50), or alternatively, skewing the production of NAbs toward IgM and IgA. In ongoing maternal infections, the compounded actions of a short interval for NAb transplacental transfer to occur, of altered IgG-NAb glycosylation patterns and/or subclasses distribution, and of lower amounts of IgG-NAb produced due to skewing of the NAb production to IgM and IgA might contribute to the very low detection of IgG-NAb in the neonates.

NK cells play a fundamental role in providing early protective immunity against viral infections, including SARS-CoV-2 (23, 70). Enhanced NK cell function has been reported in human gestation (71) and NK cells have been proposed to play an important role in response to gestational influenza A virus infection (72). We found that an early immune response conveyed by anti-spike IgM antibodies and NK cells is associated with asymptomatic maternal infection. A previous study had shown that severe COVID-19 is characterized by NK cell incapacity in controlling SARS-CoV-2 infection (22). Thus, it is tempting to speculate that NK cells might play an important role not only in controlling the development of severe COVID-19 (22) but also in the asymptomatic resolution of infection. We found that maternal IL-10 production inversely correlated with NK cell frequency. IL-10 has been reported to promote NK cell antiviral responses during acute viral infection (73). Thus, it is possible that the decrease in circulating NK cell frequency is the result of IL-10–mediated activation and recruitment to the infection site. When we interpolated our results with clinical data, we remarked that in mothers with ongoing infection, delayed umbilical cord clamping led to a selective enrichment of NK cells, but not of B or CD4^+^ T cells, in the cord blood (74). Our data suggest that ongoing maternal infection prompts NK cell expansion in the placenta that can be more readily detected in the cord blood upon delayed clamping. This prompting of fetal NK cells by ongoing maternal SARS-CoV-2 infection has been described by others (8).

Consistent with previous reports (7, 8), we show that upon SARS-CoV-2 infection, pregnant women mount an inflammatory response composed of IFN-α, IL-6, IL-10, IL-18, IL-33, and MCP-1. Intriguingly, we found that while cytokines related to acute viral response returned to normal levels upon infection clearance, the inflammatory response mediated by IL-6 and IL-18 remained elevated for weeks to months past infection resolution. Sustained inflammatory activation after asymptomatic or mild infections might bring some concerns in the future neurological development of the neonate, as maternal inflammation, including that mediated by IL-6 and IL-18, has been linked to altered immune responses (75), immune-mediated diseases (76), and an increased risk of neurological disorders later in life (77, 78). This might be even more worrying, since we observed higher concentration of at least one inflammatory cytokine in the cord blood in approximately 50% of dyads. Ex vivo studies of term placentas indicate that transplacental transfer of most cytokines does not occur (79). However, other studies indicate that maternal inflammatory responses might lead to placental cytokine production (28, 29) and transfer of maternal cytokines has been documented in in vivo animal models (80).

A combination of uncommon SARS-CoV-2 viremia in pregnant women (81) and negligible placental coexpression of its canonical cell entry receptors ACE2 and TMPRSS2 (82) accounts for the low frequency of SARS-CoV-2 vertical transmission. The most accurate way to identify vertical transmission is through the detection of anti–SARS-CoV-2 IgA and IgM antibodies in cord blood. Out of 50 paired mother-neonate dyads, we detected anti-spike IgA and IgM in 1 cord blood sample, which is consistent with previous studies that reported anti-spike IgM in 0%–7.7% of cord blood samples (7, 40, 83). Curiously, our putative case of vertical transmission occurred during the second trimester and even though maternal infection had long resolved by the time of delivery, the cord blood displayed higher concentration of 8 distinct cytokines than the mother. Moreover, this cord blood also exhibited an exceptionally high IgD^–^ B cell population documenting a fetal adaptive immune response, possibly to SARS-CoV-2 infection.

Our study has some limitations. It is difficult to quantify the relative contribution of each antibody isotype to both maternal and fetal protective immunity. We did not assess whether anti-spike/RBD IgA or IgM had undergone affinity maturation and were being produced by long-lived IgA or IgM plasma cells (84, 85). Nonetheless, NAbs with little or no somatic hypermutation have been shown to potently neutralize SARS-CoV-2, indicating that extensive B cell maturation and isotype switching is not required for NAb development (10, 86).

The maternal immune response reported here might benefit the neonate in 2 ways, first by skewing the maternal immune response toward immediate viral clearance, and second by endowing the neonate with protective mechanisms to curtail horizontal viral transmission in the critical postnatal period, via the priming of IgA/IgM-NAbs transferred by the breast milk and by prompting fetal NK cell expansion.

## Methods

### Biospecimen collection.

A total of 79 peripheral and 69 cord blood samples were collected at the time of delivery from 60 SARS-CoV-2–infected pregnant women, 12 noninfected pregnant controls, and 9 from COVID-19 mRNA–vaccinated pregnant women. SARS-CoV-2–positive and –negative participants were recruited between May, 2020 and February, 2021, before vaccination had become available to the general population. Vaccinated participants were recruited between July, 2021 and February, 2022, and maternal and cord blood samples were collected on average 73.9 days after inoculation with the second vaccine dose. Nasopharyngeal swabs were obtained for all study participants upon hospital admission and in routine consultations. Pregnant women were mandatorily tested for SARS-CoV-2 by PCR in the context of monthly health provision, which allowed us to determine both the time of viral infection and resolution. Nasopharyngeal swabs of the babies were collected, whenever possible, within 12 hours after birth. In a subgroup of participants, at their request, the clamping of the umbilical cord was delayed until the cord stopped pulsating (delayed clamping). All women underwent clinical evaluation of vital signs and symptoms, laboratory analysis, and radiological chest assessment at the discretion of physicians. Therapeutic management was consequently tailored according to clinical findings and national guidelines. Demographic and clinical characteristics are detailed in Supplemental Tables 1–3. Ethnicity attribution was made by the participants. Cord blood was obtained through venipuncture of the cord vein. Blood samples were immediately processed.

### Exclusion criteria.

In total, we recruited 63 pregnant women suspected of SARS-CoV-2 infection. Three participants were excluded from the study due to loss of biospecimens’ integrity. One participant was excluded from the control group due to prior exposure to SARS-CoV-2.

### Peripheral and cord blood mononuclear cell isolation.

Peripheral and cord blood samples were collected in EDTA tubes. PBMCs and cord blood mononuclear cells (CBMCs) were isolated by density gradient centrifugation (Biocoll, Merck Millipore, L-6715) (3, 87), cryopreserved in 10% DMSO in FBS, and stored at –80°C until subsequent analysis. Plasma samples were carefully removed from the cellular fraction and stored at –80°C until further analysis.

### ELISA.

Antibody binding to SARS-CoV-2 trimeric spike protein or its RBD was assessed by a previously described in-house ELISA assay (88) based on the protocol by Stadlbauer et al. (89). Briefly, 96-well plates (Nunc) were coated overnight at 4°C with 0.5 μg/mL trimeric spike or RBD. After blocking with 3% BSA diluted in PBS with 0.05% Tween, 1:50 diluted plasma was added and incubated for 1 hour at room temperature. Plates were washed and incubated for 30 minutes at room temperature with HRP-conjugated anti–human IgA, IgG, and IgM antibodies (Abcam; ab97225, ab97215, and ab97205) diluted 1:25,000 in 1% BSA/0.05% Tween–PBS. Plates were washed and incubated with TMB substrate (BioLegend, 421101), stopped by adding phosphoric acid (Sigma-Aldrich, P5811), and read at 450 nm. The cutoff for plasma sample positivity was defined as the mean of OD_450_ values from negative controls plus 3 times the standard deviation (88). Endpoint titers were established using a 3-fold dilution series starting at 1:50 and ending at 1:109,350 and defined as the last dilution before the signal dropped below an OD_450_ of 0.15. This value was established using plasma from prepandemic samples collected from participants not exposed to SARS-CoV-2 (88). For samples that exceeded an OD_450_ of 0.15 at last dilution (1:109,350), endpoint titer was determined by interpolation (90). The transplacental IgG transfer ratios were determined as the ratio of the IgG OD_450_ nm value in the cord blood versus the IgG OD_450_ nm value in maternal circulation at the same plasma dilution. As previously described (88), in each assay we used 6 internal calibrators from 2 high-, 2 medium-, and 2 low-antibody producers who had been diagnosed with COVID-19 through RT-PCR of nasopharyngeal and/or oropharyngeal swabs. As negative controls, we used prepandemic plasma samples collected prior to July, 2019.

### Purification of IgA, IgG, and IgM.

The IgM fraction was purified using a home-made IgM resin. Briefly, the IgM antibody (SICGEN ANTIBODIES, AB0405-500) was covalently bonded to glyoxal agarose beads resin (ABT, 6BCL-GM3) according to the manufacturer’s instructions. IgA and IgG from plasma samples were purified through Peptide M/Agarose (Invivogen, 6457-43-01) or Protein G (Thermo Fisher Scientific, 20398), respectively, according to the manufacturers’ instructions. Briefly, 100 μL of plasma was incubated with 200 μL of Peptide M/Agarose, Protein G, or IgM resin for 20 minutes. The resins/beads were washed 5 times with wash buffer (10 mM sodium phosphate, 150 mM sodium chloride; pH 7.2) and eluted in 100 μL fractions with 0.1 M glycine pH 2.76. The pH of the collected fractions was adjusted to 7 with 1 M Tris (pH 8.83). All steps were carried out at 4°C. Western blotting was performed according to standard procedures. Secondary antibodies used were from Abcam, diluted 1:5000: goat anti–human IgA alpha chain HRP (ab97215), goat anti–human IgG Fc HRP (ab97225), and goat anti–human IgM mu chain HRP (ab97205). Protein bands were imaged using ECL on a GE Amersham Imager 680.

### Production of ACE2-expressing 293T cells.

Production of 293T cells stably expressing human ACE2 receptor was done as previously described (91). Briefly, for production of VSV-G pseudotyped lentiviruses encoding human ACE2, 293T cells (provided by Paul Digard, Roslin Institute, University of Edinburgh, United Kingdom) were transfected with pVSV-G, psPAX2, and pLEX-ACE2 using jetPRIME (Polyplus), according to the manufacturer’s instructions. Lentiviral particles in the supernatant were collected after 3 days and were used to transduce 293T cells. Three days after transduction, puromycin (Merck, 540411) was added to the medium, to a final concentration of 2.5 μg/mL, to select for infected cells. Puromycin selection was maintained until all cells in the control plate died and then reduced to half. The 293T-Ace2 cell line was passaged 6 times before use and maintained in culture medium supplemented with 1.25 μg/mL puromycin.

### Production of spike-pseudotyped lentivirus.

To generate spike-pseudotyped lentiviral particles, 6 × 10^6^ 293ET cells (from Colin Adrain, Gulbenkian Institute of Science, Oeiras, Portugal) were cotransfected with 8.89 mg pLex-GFP reporter, 6.67 mg psPAX2, and 4.44 mg pCAGGS-SARS-CoV-2-S_trunc_ D614G, using jetPRIME according to the manufacturer’s instructions. The virus-containing supernatant was collected after 3 days, concentrated 10- to 20-fold using a Lenti-X Concentrator (Takara, 631231), aliquoted, and stored at –80°C. Pseudovirus stocks were titrated by serial dilution and transduction of 293T-Ace2 cells. At 24 hours after transduction, the percentage of GFP-positive cells was determined by flow cytometry, and the number of transduction units per mL was calculated.

### Neutralization assay.

Heat-inactivated plasma was 4-fold serially diluted and then incubated with spike-pseudotyped lentiviral particles for 1 hour at 37°C. The mix was added to a preseeded plate of 293T-Ace2 cells, with a final MOI of 0.2. At 48 hours after transduction, the fluorescence signal was measured using the GloMax Explorer System (Promega). The relative fluorescence units were normalized to those derived from the virus control wells (cells infected in the absence of plasma), after subtraction of the background in the control groups with cells only.

### Immunophenotyping of maternal and cord blood leukocytes.

For immunophenotyping of B, T, and NK cells, cryopreserved PBMCs and CBMCs were rested for 1 hour at 37°C and then stained with the fixable viability dye eFluor 506 (Invitrogen, 65-0866-14) and surface labeled with the following antibodies from BioLegend: anti-CD3 (300424/UCHT1), anti-CD4 (344666/SK3), anti-CD69 (310910/FN50), anti-CXCR5 (356904/J252D4), anti-CCR6 (353432/G034E3), anti-CD19 (363026/SJ25C1), anti-IgD (348250/IA6-2), and anti-CD56 (318348/HCD56). Cells were washed, fixed with 1% paraformaldehyde, and acquired in a FACSAria III (BD Biosciences) and analyzed with FlowJo v10.7.3 software (Tree Star).

### Luminex.

Plasma samples were thawed and tested in the 13-plex LegendPlex Human Inflammation panel 1 (BioLegend, 740809) to quantify levels of IL-1β, IFN-α2, IFN-γ, TNF-α, MCP-1, IL-6, IL-8, IL-10, IL-12p70, IL-17A, IL-18, IL-23, and IL-33. The assay was performed according to the manufacturer’s instructions and was modified by using half of the amount of all reagents. All plasma samples were diluted 2-fold with assay buffer, and sample concentrations were calculated according to the dilution factor. Briefly, 12.5 μL of diluted plasma or standard and 12.5 μL of mixed beads were added to each well and incubated for 2 hours. The plate (V-bottom 96-well plate) was washed twice with 100 μL of wash buffer. Samples and standards were incubated with 12.5 μL of detection antibody for 1 hour followed by a 30-minute incubation with 12.5 μL of Streptavidin-PE. The plate was washed once, and samples were resuspended in 75 μL of wash buffer. All incubation steps were performed at room temperature and protected from the light. Samples were acquired in a FACSCanto (BD Biosciences) and analyzed with LegendPlex software v8.0 for Windows (BioLegend).

### Statistics.

Statistical analysis was performed by using GraphPad Prism v9.00. First, we tested the normality of the data by using the D’Agostino & Pearson normality test, by checking skewness and kurtosis values and visual inspection of data. Then, if the samples followed a normal distribution, we chose the appropriate parametric test; otherwise, the nonparametric counterpart was chosen. In 2-group comparisons, for paired data the Wilcoxon’s matched-pairs signed-rank test and paired *t* test were used; for unpaired data, Mann-Whitney test and the unpaired *t* test were used. For multiple group comparison, ordinary 1-way ANOVA with Holm-Šidák multiple-comparison test or Kruskal-Wallis tests with Dunn’s multiple-comparison test were used as indicated in figure legends. Spearman’s and Pearson’s correlation tests were used in correlation analysis as described. A *P* value of less than 0.05 was considered significant: *P < 0.05, **P < 0.01, ***P < 0.001, ****P < 0.0001. The cord/maternal ratios for inflammatory cytokines were imported into a Microsoft Excel spreadsheet and analyzed in R v2022.2.3.0 (https://cran.rstudio.com/) to generate matrices with the ggplot package. Polar plots were generated in Origin 2022 (https://www.originlab.com/2022). The NT_50_, defined as the reciprocal of the dilution at which infection was decreased by 50%, was determined using 4-parameter nonlinear regression (least-squares regression without weighting; constraints: bottom = 0). To measure the magnitude of the difference, the effect size was calculated as described previously (92, 93). For paired *t* tests: Cohen’s *d* is small if less than 0.3, medium if 0.3 or greater, or large if 0.8 or greater. For Wilcoxon’s and Mann-Whitney tests: correlation coefficient *r* is small if less than 0.3, medium if 0.3 or greater, or large if 0.5 or greater. For Kruskal-Wallis and ordinary 1-way ANOVA: η^2^ is small if less than 0.01, medium if 0.06 or greater, or large if 0.14 or greater. Effect size values are reported in figure legends and are labeled as ^+^ for small, ^++^ for medium, and ^+++^ for large.

### Study approval.

All participants provided informed consent and all procedures were approved by the ethics committees of Centro Hospitalar de Lisboa Central (859/2020) and of NOVA Medical School (112/2021/CEFCM), in accordance with the provisions of the Declaration of Helsinki and the Good Clinical Practice guidelines of the International Conference on Harmonization.

### Data availability.

All the Supporting Data Values can be accessed in the online supplemental data files.

## Author contributions

JG, MM, and MA designed and performed experiments and analyzed the data. NC, FS, JG, and HS enrolled the participants and collected demographic data. JCN, FF, and DAS helped with some experiments. JSR provided technical support. MJA, CA, and CP provided critical expertise. HS conceptualized the study, designed experiments, analyzed the data, supervised the project, and wrote the manuscript. All authors discussed the results and commented on the manuscript.

## Supplementary Material

Supplemental data

Supporting data values

## Version 1 07/25/2023

In-Press Preview

## Version 2 09/08/2023

Electronic publication
