# Supplementary material for: Balance between maternal antiviral response and placental transfer of protection in gestational SARS-CoV-2 infection
Source: JCI Insight. 2023 Sep 8;8(17):e167140. doi: 10.1172/jci.insight.167140 (PMC10544212; doi:10.1172/jci.insight.167140)
Supplement: Supplemental data [file jciinsight-8-167140-s009.pdf]

Figure S1

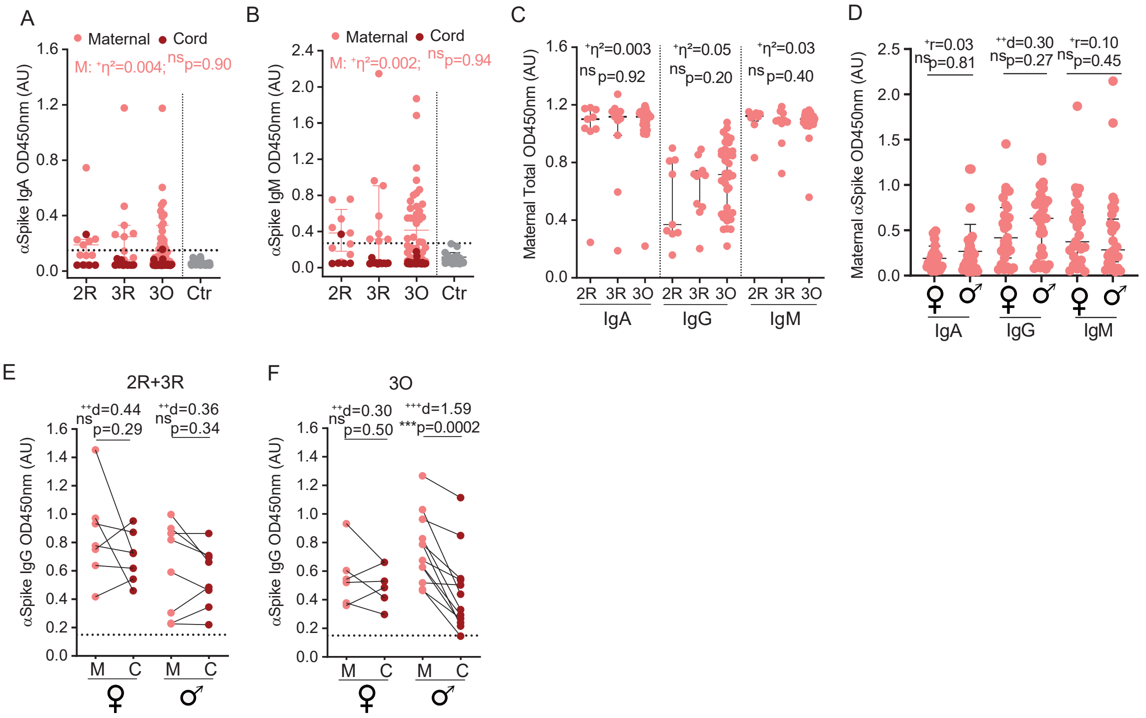

Figure S1- Antibody levels in maternal:neonate dyads segregated by neonate sex and symptomatology.

(**A**) Anti-spike IgA in maternal and cord blood of recovered (2R, 3R) and ongoing (3O) infection (CoV-2+, n=60) and non-infected controls (CoV-2-, n=12), measured by absorbance at 450 nm (OD450) in arbitrary units (AU).

(**B**) Anti-spike IgM as in (A).

(**C**) Total IgA, IgG and IgM antibody levels segregated by gestational period of infection (n=58).

(**D**) Anti-spike IgA, IgG and IgM in maternal CoV-2+ plasma according to neonate sex (n=56).

(**E**) Paired dyads analysis of anti-spike IgG in recovered (2R+3R) maternal infection according to neonate sex (n=15)

(**F**) Paired dyads analysis of anti-spike IgG in ongoing (3O) maternal infection according to neonate sex (n=18).

Dashed line indicates assay cutoff. Data represents mean ± SD for parametric tests, or median ± IQR for non-parametric tests. p values ***p < 0.001; ns, not significant were determined by Kruskal-Wallis test (A-C), unpaired t test (D), Mann-Whitney test (D) and parametric paired t test (E, F). Effect sizes were determined by η2 = eta-squared (A-C), d = Cohen's d (D-F) and r = correlation coefficient r (D).

Figure S2

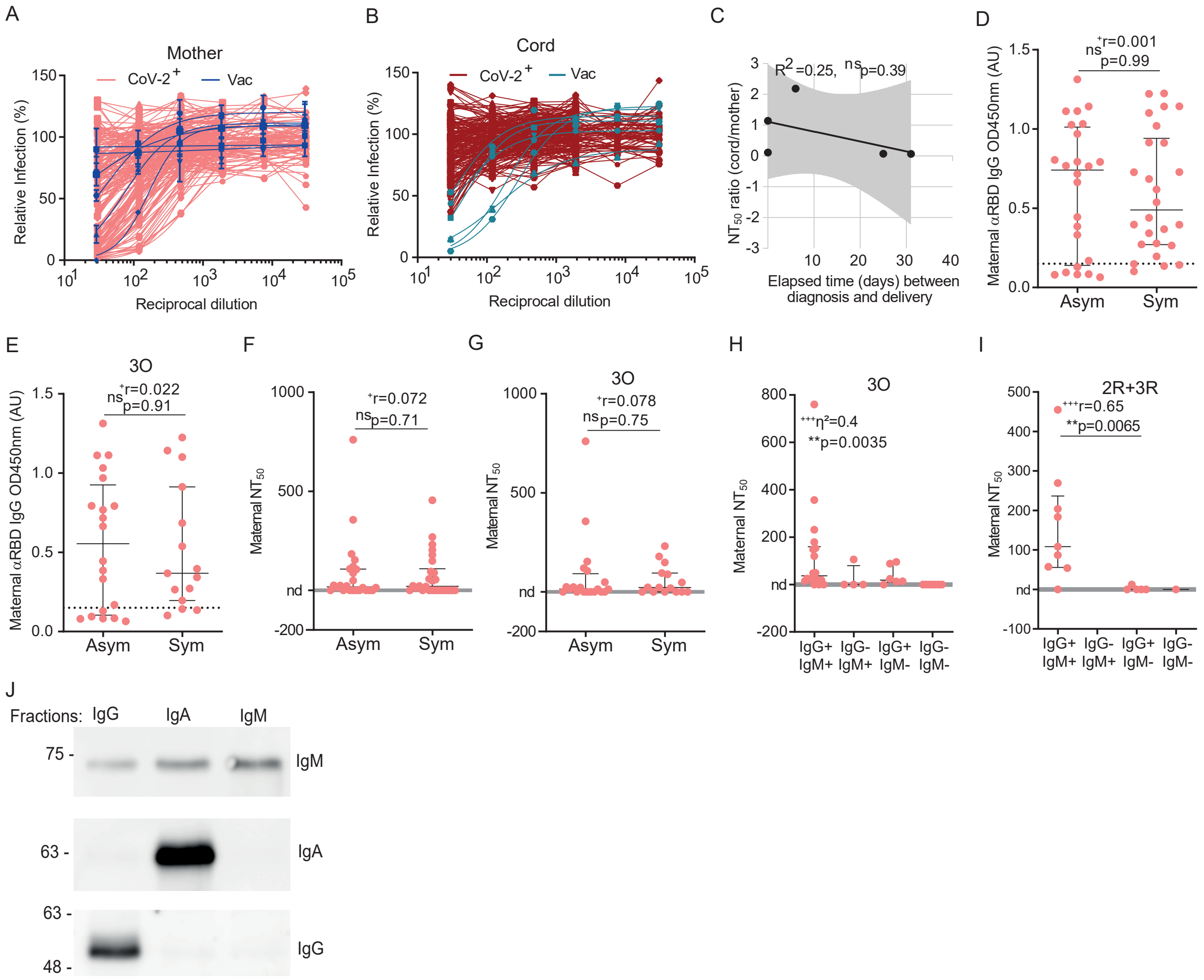

Figure S2- Neutralizing antibody levels in maternal:neonate dyads.

(**A**) Neutralization curves for maternal plasma from SARS-CoV-2 positive (CoV-2+, n = 50) and vaccinated (Vac, n = 6) individuals.

(**B**) Neutralization curves for cord plasma from SARS-CoV-2 positive (CoV-2+, n = 50) and vaccinated (Vac, n = 6) individuals.

(**C**) Correlation between NT50 transfer ratio and elapsed time between diagnosis and delivery (n=5).

(**D**) Anti-RBD IgG in asymptomatic (Asym) and symptomatic (Sym) CoV-2+ participants (n=50).

(**E**) Anti-RBD IgG in asymptomatic (Asym) and symptomatic (Sym) in ongoing maternal infections (n=35).

(**F**) NT50 in asymptomatic (Asym) and symptomatic (Sym) maternal infection (n = 50).

(**G**) NT50 in asymptomatic (Asym) and symptomatic (Sym) in ongoing maternal infections (3O, n = 35).

(**H**) Maternal NT50 in function of the presence of anti-spike IgG and/or IgM antibodies in ongoing infections (3O, n = 35).

(**I**) Maternal NT50 in function of the presence of anti-spike IgG and/or IgM antibodies in recovered infections (2R+3R, n = 15).

(**J**) Western blot for detection of IgM, IgA and IgG immunoglobulins in purified IgG, IgA and IgM fractions.

Dashed line indicates assay cutoff. nd: not detectable. Data represents median ± IQR for non-parametric tests. p values **p < 0.01; ns, not significant were determined by Pearson correlation (C), Mann-Whitney test (D-G, I) and Kruskal-Wallis test (H). Effect sizes were determined by r = correlation coefficient r (D-G, I) and η2 = eta-squared (H).

Figure S3

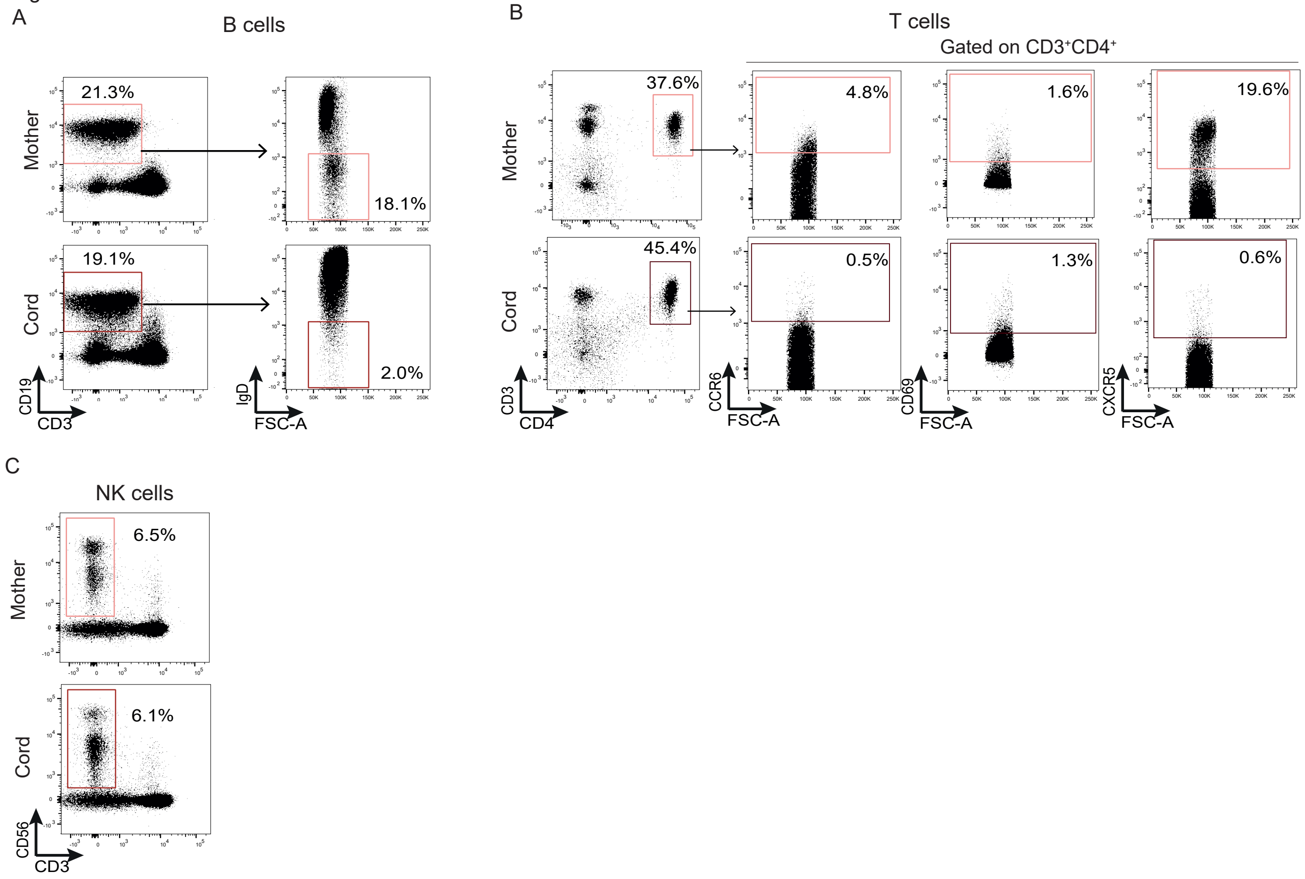

Figure S3- Flow cytometric gating strategy.

(**A-C**) Gating strategy for (A) CD3-CD19+IgD- B cells; (B) CD3+CD4+CCR6+, CD3+CD4+CD69+ and CD3+CD4+CXCR5+ T cells; (C) CD3-CD56+ NK cells in maternal and cord blood samples.

Figure S4

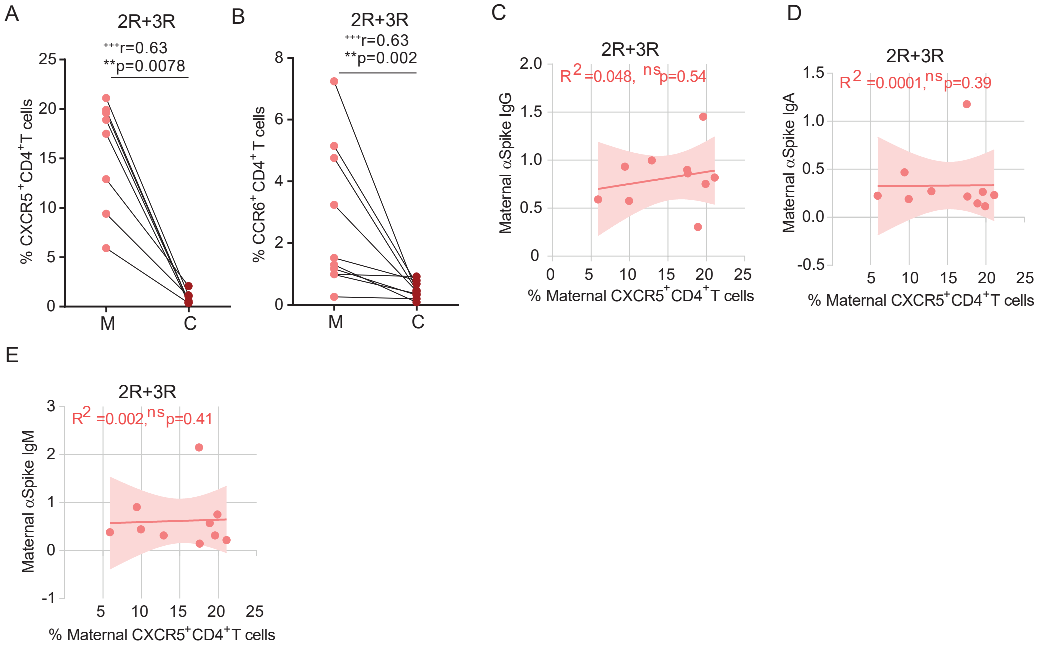

Figure S4- Long-term expansion of CXCR5+CD4+ T cells in maternal SARS-CoV-2 infection.

(**A**) Paired dyad analysis of the frequency of CXCR5+CD4+ T cells in SARS-CoV-2 recovered participants (2R+3R, n=8).

(**B**) As in (A) for CCR6+CD4+ T cells (2R+3R, n=10).

(**C**) Correlation between anti-spike IgG and the frequency of CXCR5+CD4+ T cells in recovered maternal infection (2R+3R, n = 10).

(**D**) As in (C) for maternal anti-spike IgA levels (2R+3R, n = 10).

(**E**) As in (C) for maternal anti-spike IgM levels (2R+3R, n = 10).

p values **p < 0.01; ns, not significant were determined by non-parametric paired Wilcoxon test (A, B), Pearson correlation (C) and Spearman correlation (D, E). Effect sizes were determined by r = correlation coefficient r (A, B).

Figure S5

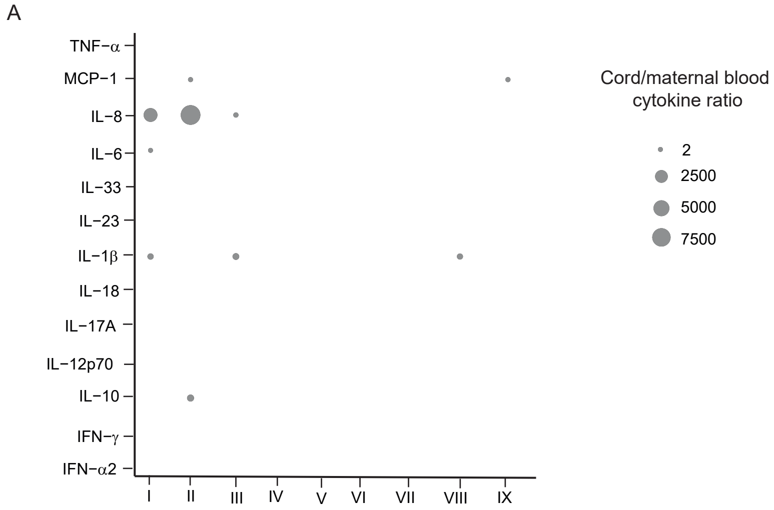

Figure S5- Inflammatory profile in the plasma of non-infected non-vaccinated mother:neonate dyads, in uninfected and non-vaccinated participants.

(**A**)- Ratio between the concentration of the cytokines IFN-α2, IFN-γ, IL-17A, IL-1β, IL-33, IL-6, TNF-α, IL-18, IL-10, IL-12p70, IL-23, IL-8, and MCP-1 in the cord blood and in the maternal blood in non-infected and unvaccinated pregnant women (n = 9).

Table S1: Demographic and clinical data of pregnant women infected with SARS-CoV-2 (CoV-2^{+}) categorized between asymptomatic and symptomatic.

	**Asymptomatic (n = 30)**	**Symptomatic (n = 30)**
**Age categories n (%)**		
18-26	9 (30%)	7 (23.3%)
27-33	11 (36.7%)	15 (50%)
34-40	10 (33.3%)	8 (26.7%)
**Ethnicity n (%)**		
Black	9 (30%)	4 (13.3%)
Asian	9 (30%)	3 (10%)
White	11 (36.7%)	21 (70%)
Romani	0	1 (3.3%)
Unretrieved	1 (3.3%)	1 (3.3%)
**Comorbidities n (%)**		
Anaemia	3 (10%)	1 (3.3%)
Cardiovascular diseases	2 (6.7%)	1 (3.3%)
Endocrine System	2 (6.7%)	2 (6.7%)
Pulmonary diseases	0	4 (13.3%)
Obesity	3 (10%)	4 (13.3%)
Others	2 (6.7%)	3 (10%)
None	18 (60%)	15 (50%)
**Delivery Mode n (%)**		
Vaginal	9 (30%)	10 (33.3)
Caesarean	17 (56.7%)	13 (43.3%)
Forceps	3 (10%)	7 (23.3)
Unretrieved	1 (3.3%)	0
**Cord Clamping n (%)**		
Delayed	14 (46.7%)	14 (46.7%)
Immediate	10 (33.3%)	12 (40%)
Unknown	6 (20%)	4 (13.3%)
**SARS-CoV-2 positive PCR in mothers**	100%	100%
**SARS-CoV-2 PCR in new-born n (%)**		
Negative	26 (86.7%)	20 (66.7%)
Positive	2 (6.7%)	1 (3.3%)
No PCR	2 (6.7%)	9 (30%)
**Sex new-born n (%)**		
Female	14 (46.7%)	14 (46.7%)
Male	16 (53.3%)	14 (46.7%)
Unretrieved	0	2 (6.7%)

**Weight new-born, g, n (%)**		
<2500	2 (6.7%)	3 (10%)
2501-3000	13 (43.3%)	9 (30%)
3001-4000	14 (46.7%)	15 (50%)
>4000	1 (3.3%)	2 (6.7%)
Unretrieved	0	1 (3.3%)
**Diagnostic Trimester n (%)**		
Second (recovered at delivery) - 2R	3 (10%)	6 (20%)
Third (recovered at delivery) - 3R	2 (6.7%)	9 (30%)
Third (ongoing infection at delivery) - 3O	25 (83.3%)	15 (50%)
**Sick Contact n (%)**		
Yes	10 (33.3%)	9 (30%)
No	18 (60%)	21 (70%)
Unknown	2 (6.7%)	0
**Invasive Ventilation n (%)**		
Yes	0	1 (3.3%)
No	29 (96.7%)	29 (96.7%)
Unknown	1 (3.3%)	0
**Oxygen Support n (%)**		
Yes	0	4 (13.3%)
No	29 (96.7%)	26 (86.7%)
Unknown	1 (3.3%)	0
**Imaging of Pneumonia n (%)**		
Yes	0	4 (13.3%)
No	28 (93.3%)	26 (86.7%)
Unknown	2 (6.7%)	0
**COVID-19 hospitalization n (%)**		
Yes – intensive care	0	1 (3.3%)
Yes – no intensive care	0	3 (10%)
No	28 (93.3%)	26 (86.7%)
Unknown	2 (6.7%)	0

Table S2: Demographic and clinical data of pregnant women negative for SARS-CoV-2 (CoV-2^{-}).

**SARS-CoV-2 negative PCR in mothers**	n=12
**Age Categories n (%)**	
18-26	2 (16.7%)
27-33	5 (41.7%)
34-40	5 (41.7%)
**Ethnicity n (%)**	
Black	3 (25%)
Asian	1 (8.3%)
White	7 (58.3%)
Unretrieved	1 (8.3%)
**Delivery Mode n (%)**	
Vaginal	4 (33.3%)
Caesarean	7 (58.3%)
Forceps	1 (8.3%)
**Sex new-born n (%)**	
Female	3 (25%)
Male	9 (75%)
**Weight new-born, g, n (%)**	
<2500	1 (8.3%)
2501-3000	1 (8.3%)
3001-4000	10 (83.3%)

Table S3: Demographic and clinical data of pregnant women vaccinated with mRNA COVID-19 BNT162b vaccine.

**Vaccinated mothers with mRNA COVID-19 vaccine**	n=9
**Age Categories n (%)**	
18-26	0
27-33	4 (44.4%)
34-40	5 (55.6%)
**Ethnicity n (%)**	
White	9 (100%)
**Delivery Mode n (%)**	
Vaginal	2 (22.2%)
Caesarean	3 (33.4%)
Forceps	2 (22.2%)
Unretrieved	2 (22.2%)
**Sex new-born n (%)**	
Female	3 (33.4%)
Male	4 (44.4%)
Unretrieved	2 (22.2%)
**Weight new-born, g, n (%)**	
<2500	0
2501-3000	2 (22.2%)
3001-4000	4 (44.4%)
>4000	1 (11.2%)
Unretrieved	2 (22.2%)
